# Incidence of Severe Chronic Neutropenia in South Korea and Related Clinical Manifestations: A National Health Insurance Database Study

**DOI:** 10.3390/medicina56060262

**Published:** 2020-05-27

**Authors:** Nuri Lee, Boung Chul Lee

**Affiliations:** 1 Department of Laboratory of Medicine, Kangnam Sacred Heart Hospital, Hallym University, College of Medicine, Seoul 07441, Korea; nurilee822@hallym.or.kr; 2 Department of Psychiatry, Hangang Sacred Heart Hospital, Hallym University College of Medicine, Seoul 07247, Korea; 3 Health Insurance Review and Assessment Service, Seoul 07247, Korea

**Keywords:** neutropenia, severe chronic, epidemiology, incidence, myelodysplastic syndromes, leukemia

## Abstract

*Background and objectives*: Severe chronic neutropenia (SCN) is a condition in which absolute neutrophil counts remain at a low level (under 500/µL) over months or years. Because of the rare onset of SCN, its epidemiology, prognosis, and clinical manifestations have not yet been fully understood. In particular, large-cohort studies in Asian countries are still insufficient. Therefore, in this study, national health insurance data was used to investigate the epidemiologic features and prognosis of SCN in South Korea. *Materials and Methods*: The data from the Health Insurance Review and Assessment database recorded between 1 January 2011 and 31 December 2015 were explored. SCN was defined based on the ICD-10 code, registry of benefit extension policy, and inclusion criteria of the study. After identifying patients with SCN, annual incidence and their co-morbidities were analyzed. *Results*: Among the initially identified patients with severe neutropenia (N = 2145), a total of 367 patients had SCN and were enrolled. The annual incidence rate of SCN ranged from 0.12 to 0.17 per 100,000 person-year (PY) during the study period. The highest incidence was observed in pediatric patients aged between 0 to 9 years (N = 156), followed by women in their fifties (N = 43). The total incidence rate was 0.17 in females and 0.12 in males (Relative risk (RR): 1.43, 95%, CI: 1.16–1.76). The most common accompanying condition was mild respiratory infection, but about 3.2% of patients progressed to hematologic malignancy after an average of 2.4 years. *Conclusions*: This nationwide population-based epidemiological study showed that incidence of SCN is higher in pediatrics and middle-aged women. As progression to hematologic malignancy was significantly higher in the age of in 45–49 years old, careful follow-up is necessary in this group. However, since this study lacks the molecular information, these finding need to be interpreted with great caution.

## 1. Introduction

Severe chronic neutropenia (SCN) is a condition in which absolute neutrophil counts remain under 500/µL for months or years. This acute form of severe primary neutropenia is caused by cytotoxic drugs or infectious agents, whereas chronic forms arise from congenital, idiopathic, and autoimmune factors [1,2]. Generally, neutropenia caused by previous hematologic disease, systemic autoimmune disease, and infectious disease is excluded from SCN [1,2,3,4]. 

Neutropenia is a rather rare disorder and has a wide range of disease patterns. Most of the patients with neutropenia have mild symptoms, but some neutropenic patients with chronic and severe forms develop recurrent chronic fever and life-threatening infection due to long-term immunodeficiency. Moreover, a few of them develop bone marrow diseases such as myelodysplastic syndrome (MDS) or leukemia [5,6,7]. Due to the low incidence of SCN, its epidemiology, prognosis, and clinical manifestations have not yet been fully understood. Research based on the Severe Chronic Neutropenia International Registry (SCNIR) has helped to expand the understanding of chronic neutropenia [2,8,9]. The etiology of SCN is being actively researched, including genomic studies by targeted next generation sequencing; exome sequencing studies [10,11,12,13,14]; studies of immunologic parameters [5,8,15]; and cytokines/chemokines [16,17]. 

Few case reports regarding SCN have been published on Asian populations [18,19,20]. As SCN may have racial specificity, and differences in disease distribution by age and sex have been identified in previous studies, efforts to identify epidemiologic features in various backgrounds are of high importance for understanding this disease [2,4,8,21]. Therefore, the aim of this study was to investigate the annual incidence of SCN and its clinical manifestations in Koreans on the basis of domestic health insurance data.

## 2. Materials and Methods

### 2.1. Sources of Data and Data Selection

Ethical approval for the research protocol was provided by the Institutional Review Board of the Hangang Sacred Heart Hospital, Hallym University College of Medicine, Seoul, Korea (2018-071, Ethical approve date: 2018.12.05).

The Health Insurance Review and Assessment (HIRA) database and Korean population data from the Korean Statistical Information Service (KOSIS) were used. The HIRA data were obtained from a payment request form generated each time a hospitalized patient or outpatient visits a medical institution. The HIRA database provides information on all insurance claims including patient demographics, diagnosis coded according to ICD-10, dates of hospital visits, procedures, and drug prescriptions for about 97.0% of the population of Korea [22,23]. The HIRA data were reviewed and filtered to determine the total SCN population. Patients diagnosed with neutropenia (D70) and designated as patients with benefit extension policy for rare disease (V108, congenital agranulocytes, or cyclic/chronic neutropenia over 1 month (neutrophil count < 500/µL)) from January 2011 to December 2015 were entered into the database (N = 2145). We extracted the first claim date, the total number of claims, date when each patient was diagnosed with neutropenia (D70), and the associated diseases. To screen only chronic neutropenia patients, patients with a follow-up period of at least 1 year and a total number of claims of at least 5 were selected (N = 422). The exclusion criteria are as follows: previously diagnosed disease that may cause neutropenia, namely, cytotoxic treatment and hematologic diseases, as well as conditions known to be associated with neutropenia including autoimmune diseases, viral disease, and immunodeficiency associated disorder [1,6,8]. A total of 367 SCN patients were identified based on these exclusion criteria. 

### 2.2. Estimation of the Incidence of SCN and Associated Clinical Features

The total number of Koreans in 2011 to 2015 and the distribution of population by age and gender were obtained through the KOSIS. The incidence rate was calculated by dividing the number of new cases per year by the total population. Differences in incidence by age and sex, and significance of gender and age differences were also identified. Additional co-morbidities that occurred from 2011 to 2017 were investigated to analyze the statistical significance of the effects of the most common co-morbid diseases; the statistical significance of the gender and age differences was also determined. The incidence of inflammatory diseases and hematologic malignancies such as MDS and acute leukemia, as well as mortality were investigated. The date of each claim was extracted to analyze the statistical significance of disease occurrence and progression by gender and age. Only cases with an infectious disease and hematologic malignancy following SCN diagnosis were included in associated clinical features.

### 2.3. Statistics

All statistical analyzes were performed using SPSS ver. 16 (SPSS Inc., Chicago, IL, USA) and Medcalc ver. 12 (Medcalc software, Mariakerke, Belgium). Chi-square test was used to compare the incidence of MDS or leukemia and mortality according to the sex and age group. Fisher exact test was used when the number was less than five. Statistical significance was defined as 95% confidence intervals (CIs) that did not contain 1 and *p* < 0.05.

## 3. Results

### 3.1. Annual Incidence of SCN in Korea in 2011–2015

**Figure 1:**
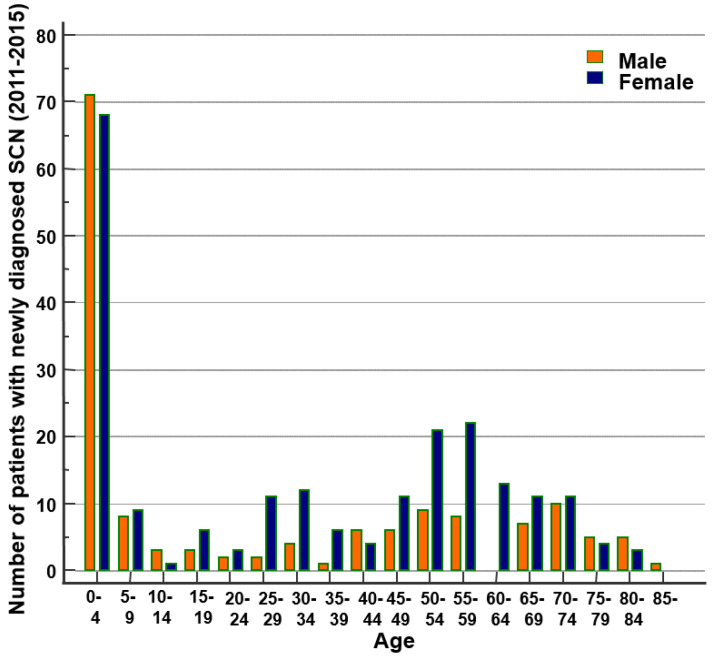
Age distribution of newly diagnosed severe chronic neutropenia (SCN) in Korea (2011–2015).

From 2011 to 2015, a total of 367 patients were diagnosed with SCN, with an average of 73.4 patients per year. The mean age at onset was 31.4 years (range: 1–92, 95% CI: 28.5–34.3); SCN occurred in 151 males (41.1%) and 216 females (58.9%). The annual incidence rate of SCN ranged from 0.12 to 0.17 per 100,000 person-year (PY) during the study period (Table 1). We compared the incidence of SCN according to age and gender. The highest incidence was observed in pediatric patients aged 0 to 9 years (N = 156), followed by women in their fifties (N = 43). The total incidence rate was 0.17 in females and 0.12 in males (relative risk (RR): 1.43, 95%, CI: 1.16–1.76, Figure 1, Table 2). There was no statistically significant difference in the incidence rate between males and females from 0 to 19 years of age. The greatest difference in incidence between males and females was between 20 and 39 years of age. The average incidence rate per 100,000 PY for 20–29-year-old patients was 0.04 for males and 0.18 for females (RR: 3.85, 95% CI: 1.27–11.69), respectively, and 0.05 and 0.18 for 30–39-year-old patients (RR: 3.75, 95% CI: 1.39–10.10).

### 3.2. Clinical Features and Associated Infectious Complications

**Figure 2:**
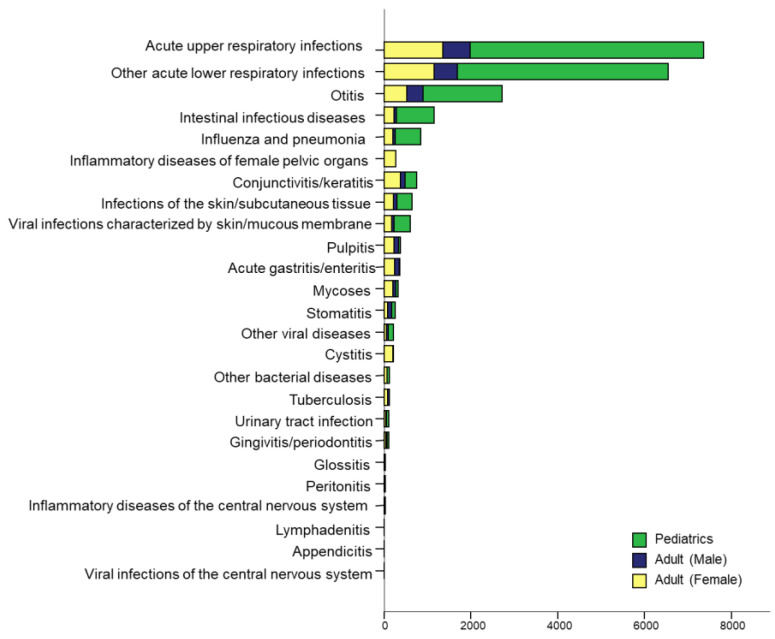
Infectious complications in patients with severe chronic neutropenia stratified by pediatric patients, as well as male and female adults.

The average number of hospital visits by SCN patients was 29.4 (range: 5–306.0, 95% CI: 26.0–32.9), and the average time from the first claim to the last claim was 34.1 months (range: 10.1–82.2 months, 95% CI: 32.4–35.7). The incidence of infections associated with SCN was analyzed separately in pediatric patients, adult males, and adult females through the number of claims (Figure 2). The total number of claims related to inflammation was highest in pediatric patients, followed by female and male patients. Acute upper respiratory tract infection (URTI) was most frequent, followed by lower respiratory tract infection (LRTI), otitis, intestinal infectious disease, influenza, and conjunctivitis. Infections of the skin and subcutaneous tissue, viral infections of the skin or mucous membrane, pulpitis, acute gastritis or enteritis, mycoses, and stomatitis were frequently encountered. Other bacterial or viral diseases including tuberculosis, urinary tract infections, gingivitis, periodontitis, and unspecified sepsis were also reported. In some patients, glossitis, peritonitis, inflammatory diseases of the central nervous system (CNS), lymphadenitis, and appendicitis were observed. In general, the pattern of accompanying inflammation was similar regardless of sex or age. In pediatric patients, the incidence of respiratory infections was overwhelmingly higher than that of other infections. Unlike in other groups, infections of pelvic organs, cystitis, and conjunctivitis showed relatively frequent occurrence in females. 

### 3.3. Hematologic Evolution to Acute Leukemia and/or MDS and Mortality

A total of 12 patients (3.2%) progressed to acute leukemia and/or MDS and one patient died during the follow-up period of the years 2011–2017. Among these patients, seven were male and five were female. There was no difference in acute leukemia and/or MDS incidence by gender. The average age at first diagnosis of SCN was 50 years (range = 11.0–73.0, 95% CI = 44.5–66.3), and after 885.3 days (range = 61.0–2192.0, 95% CI = 253.7–1404.4), SCN progressed to acute leukemia and/or MDS. When comparing the progression odds ratio of acute leukemia and/or MDS by age at the time of diagnosis of SCN, it was found that the progression was significantly higher in the age of 45–49 years compared to other age groups (progression OR = 8.01, 95% CI = 1.26–37.13, *p* = 0.014, Table 3). One patient died of acute leukemia and/or MDS during the follow-up period. The patient was diagnosed with SCN at 73 years of age, progressed to acute leukemia and/or MDS 92 days after SCN diagnosis, and died 623 days after progression.

## 4. Discussion and Conclusions

This is the first study of the epidemiologic aspects of severe and chronic neutropenia that included the entire population of South Korea. SCN is occasionally encountered in clinical settings and it is difficult to triage and warn patients because not many studies have been reported on the progress and outcome of SCN. Although the research did not include the molecular results, the health insurance data, which includes the entire population, was used to estimate the incidence rate, prognosis, and companion diseases of SCN in Korea. In this study, the mean annual incidence per 100,000 PY was 0.14, indicating that SCN is a relatively rare disease. The incidence of neutropenia was found to vary in previous studies according to population, severity of neutropenia, or etiology. In case of congenital neutropenia, the estimated prevalence per million inhabitants ranges from 0.006 to 8.5 [24,25]. A study on chronic idiopathic neutropenia revealed that the prevalence of moderate neutropenia was 1.4% of a total of 778 healthy people, with no severe neutropenia patients identified [26]. The incidence of our patient group was relatively low compared to previous studies. This may be because we focused on a specific population of patients with severe and chronic cases. Most of the previous studies calculated incidence and prevalence, including mild and moderate forms. In the two papers that studied the existing chronic neutropenia, the severe form was 9 out of 76 (11.8%) [6] and 5 out of 439 (1.1%) [27], respectively. Another reason is racial differences. According to a previous study that studied racial differences in neutropenia, there was a strong association of neutropenia in Africans [28]. Until now, studies conducted on Asian SCNs are insufficient, but it cannot be excluded that there are racial differences in incidence in Asian patients. In a study comparing country-specific prevalence with regard to congenital and idiopathic neutropenia, the Asia region was not included [24], and SCNIR had only a small number of Asians [2,4]. Research on epidemiology and prognosis in Asian countries is needed. Finally, it is possible that the difference in cohort size has affected the low incidence. Most of the previous researches have been conducted with fewer than 100 patients. In the previous study, since the estimated prevalence was often obtained based on the number of case reports [24], it is considered that it may show a difference from the number reflecting the actual entire population.

Compared with previous research, this study showed generally similar demographic distribution. In previous studies, SCN was predominantly induced at an early age, with no difference in gender-specific incidence in pediatric patients but female predominance among adult patients [8,9,26,29]. Similar results were obtained in this large cohort study. Of a total of 367 patients, a high proportion was between the ages of 0 and 9. Among pediatric patients, the number of males and females was similar. Increased incidence rates were observed in adult women, especially at the ages of 20–39 years, which had a statistically significantly higher F/M relative risk. Further research on SCN etiology is required in relation to its general prevalence in middle-aged women.

According to this study, the most common complications in SCN were mild infections including respiratory tract infection, whereas systemic and severe infections such as sepsis and meningitis were rare. When comparing the incidence of infectious disease with the normal healthy population, respiratory infections occurred more frequently in the SCN patient group, but acute fever with rash and infectious diarrheal diseases were relatively less common [30,31]. In addition, during the three- to seven-year follow-up period, 3.2% of SCN patients developed leukemia/MDS. In previous studies, the prognostic spectrum of neutropenia was varying depending on the etiology. Chronic idiopathic neutropenia has been reported to have a relatively mild prognosis [2,4,17,26,32]. However, neutropenia progress to severe forms such as hematologic malignancy in congenital neutropenia [1,6,33,34,35,36]. This study shows that severely low levels of neutrophil count during a long-term period do not necessarily lead to poor outcomes. Rather, it is suggested that different approaches depending on the causes of SCN are required to predict the prognosis.

There are several limitations to this study. First, since this study was based on claim data, the accuracy of the diagnosis could be restricted. To compensate for this, the domestic rare disease policy code was used along with the ICD code, and efforts were made to improve the accuracy by considering the claim date and the total number of visits. Secondly, we could not discriminate underlying genetic or immune disorder as the health insurance data did not show the results of laboratory tests including *ELANE*, *HAX1* gene tests or anti-neutrophil antibodies. Nevertheless, this paper is meaningful in that it provides information on the overall and basic incidence rates before genetic information is provided.

In summary, we investigated the incidence of SCN in the Korean population and found it to be 0.14 per 100,000 PY in 2010–2015. SCN is most common in children aged 0–9 years and middle-aged women, with mild form of infection, but about 3.2% of patient progress to hematologic malignancy after an average of 2.4 years. This is the first large cohort study of SCN in Korean patients, and our findings would be helpful for counseling patients with chronic and severe forms of neutropenia, providing the approximate incidence rate of disease and their prognosis.

## Figures and Tables

**Table 1:** Annual incidence rate of severe chronic neutropenia in South Korea (2011–2015).

	Total Population			New Cases			F/M Ratio of Cases	Incidence Rate per 100,000		
	All	Male	Female	All	Male	Female		All	Male	Female
2011	49,779,440	24,942,339	24,837,101	63	29	34	1.17	0.13	0.12	0.14
2012	50,004,441	25,039,557	24,964,884	81	36	45	1.25	0.16	0.14	0.18
2013	50,219,669	25,132,612	25,087,057	85	27	58	2.15	0.17	0.11	0.23
2014	50,423,955	2,5219,810	25,204,145	78	36	42	1.17	0.15	0.14	0.17
2015	51,014,947	25,585,894	25,429,053	60	23	37	1.61	0.12	0.09	0.15

**Table 2:** Average age- and sex-specific incidence of severe chronic neutropenia during the study period (2011–2015).

Age Group (Years)	Cases			Average Incidence Rate per 100,000			F/M Incidence Rate Relative Risk (95% CI)
	All	Male	Female	All	Male	Female	
0–9	156	79	77	1.36	1.33	1.38	1.03 (0.76–1.42)
10–19	13	6	7	0.08	0.07	0.08	1.28 (0.43–3.83)
20–29	18	4	14	0.11	0.04	0.18	3.85 (1.27–11.69)
30–39	23	5	18	0.11	0.05	0.18	3.75 (1.39–10.10)
40–49	27	12	15	0.12	0.10	0.14	1.30 (0.61–2.77)
50–59	60	17	43	0.31	0.17	0.44	2.56 (1.46–4.49)
60–69	31	7	24	0.29	0.15	0.41	3.20 (1.38–7.43)
70–79	30	15	15	0.38	0.46	0.32	0.73 (0.36–1.50)
>80	9	6	3	0.39	1.14	0.12	0.21 (0.05–0.85)
Total	367	151	216	0.14	0.12	0.17	1.43 (1.16–1.76)

**Table 3:** Age-specific frequency of leukemia/MDS evolution and death among severe chronic neutropenia patients.

Age Group (Years)	Leukemia/MDS Cases		
	Cases	Progression OR (95% CI)	*p* Value
10–14	1	10.46 (0.18–142.68)	0.125
25–29	1	2.59 (0.06–20.76)	0.356
40–44	1	3.47 (0.07–29.39)	0.286
45–49	3	8.01 (1.26–37.13)	0.014
50–54	1	1.03 (0.02–7.50)	>0.999
55–59	2	2.33 (0.24–11.74)	0.256
65–69	1	1.80 (0.04–13.83)	0.458
70–74	2	3.52 (0.35–18.31)	0.146

Abbreviations: MDS, myelodysplastic syndrome; OR, odds ratio; CI, confidence interval.

## References

[1] Wan C., Yu H.H., Lu M.Y., Lee J.H., Wang L.C., Lin Y.T., Yang Y.H., Chiang B.L. (2012). Clinical manifestations and outcomes of pediatric chronic neutropenia. J. Formos. Med. Assoc. Taiwan Yi Zhi.

[2] Dale D.C., Bolyard A.A., Schwinzer B.G., Pracht G., Bonilla M.A., Boxer L., Freedman M.H., Donadieu J., Kannourakis G., Alter B.P. (2006). The Severe Chronic Neutropenia International Registry: 10-Year Follow-Up Report. Support. Cancer Ther..

[3] Kyle R.A., Linman J.W. (1968). Chronic idiopathic neutropenia. A newly recognized entity?. N. Engl. J. Med..

[4] Dale D.C., Cottle T.E., Fier C.J., Bolyard A.A., Bonilla M.A., Boxer L.A., Cham B., Freedman M.H., Kannourakis G., Kinsey S.E. (2003). Severe chronic neutropenia: Treatment and follow-up of patients in the Severe Chronic Neutropenia International Registry. Am. J. Hematol..

[5] Gibson C., Berliner N. (2014). How we evaluate and treat neutropenia in adults. Blood.

[6] Fattizzo B., Zaninoni A., Consonni D., Zanella A., Gianelli U., Cortelezzi A., Barcellini W. (2015). Is chronic neutropenia always a benign disease? Evidences from a 5-year prospective study. Eur. J. Int. Med..

[7] Palmblad J., Dufour C., Papadaki H.A. (2014). How we diagnose neutropenia in the adult and elderly patient. Haematologica.

[8] Sicre de Fontbrune F., Moignet A., Beaupain B., Suarez F., Galicier L., Socie G., Varet B., Coppo P., Michel M., Pautas C. (2015). Severe chronic primary neutropenia in adults: Report on a series of 108 patients. Blood.

[9] Dale D.C., Bolyard A.A. (2017). An update on the diagnosis and treatment of chronic idiopathic neutropenia. Curr. Opin. Hematol..

[10] Donadieu J., Beaupain B., Fenneteau O., Bellanne-Chantelot C. (2017). Congenital neutropenia in the era of genomics: Classification, diagnosis, and natural history. Br. J. Haematol..

[11] Tsangaris E., Klaassen R., Fernandez C.V., Yanofsky R., Shereck E., Champagne J., Silva M., Lipton J.H., Brossard J., Michon B. (2011). Genetic analysis of inherited bone marrow failure syndromes from one prospective, comprehensive and population-based cohort and identification of novel mutations. J. Med. Genet..

[12] Touw I.P. (2015). Game of clones: The genomic evolution of severe congenital neutropenia. Hematol. Am. Soc. Hematol. Educ. Program.

[13] Mavroudi I., Papadaki H.A. (2012). Genetic associations in acquired immune-mediated bone marrow failure syndromes: Insights in aplastic anemia and chronic idiopathic neutropenia. Clin. Dev. Immunol..

[14] Dufour C., Miano M., Fioredda F. (2016). Old and new faces of neutropenia in children. Haematologica.

[15] Akhtari M., Curtis B., Waller E.K. (2009). Autoimmune neutropenia in adults. Autoimmun. Rev..

[16] Papadaki H.A., Coulocheri S., Eliopoulos G.D. (2000). Patients with chronic idiopathic neutropenia of adults have increased serum concentrations of inflammatory cytokines and chemokines. Am. J. Hematol..

[17] Papadaki H.A., Palmblad J., Eliopoulos G.D. (2001). Non-immune chronic idiopathic neutropenia of adult: An overview. Eur. J. Haematol..

[18] Shim Y.J., Kim H.J., Suh J.S., Lee K.S. (2011). Novel ELANE gene mutation in a Korean girl with severe congenital neutropenia. J. Korean Med. Sci..

[19] Shu Z., Li X.H., Bai X.M., Zhang Z.Y., Jiang L.P., Tang X.M., Zhao X.D. (2015). Clinical characteristics of severe congenital neutropenia caused by novel ELANE gene mutations. Pediatr. Infect. Dis. J..

[20] Zou T., Deng J., Shu M., Guo Q., Miao R., Wan C.M., Ning G., Zhu Y. (2018). Novel Gene Mutation in a Chinese Boy with Severe Congenital Neutropenia. Indian J. Pediatr..

[21] Donadieu J., Leblanc T., Bader Meunier B., Barkaoui M., Fenneteau O., Bertrand Y., Maier-Redelsperger M., Micheau M., Stephan J.L., Phillipe N. (2005). Analysis of risk factors for myelodysplasias, leukemias and death from infection among patients with congenital neutropenia. Experience of the French Severe Chronic Neutropenia Study Group. Haematologica.

[22] Koo B.K., Lee C.H., Yang B.R., Hwang S.S., Choi N.K. (2014). The incidence and prevalence of diabetes mellitus and related atherosclerotic complications in Korea: A National Health Insurance Database Study. PLoS ONE.

[23] Lee H.S., Lee H.S., Shin H.Y., Choi Y.C., Kim S.M. (2016). The Epidemiology of Myasthenia Gravis in Korea. Yonsei Med. J..

[24] Donadieu J., Beaupain B., Mahlaoui N., Bellanne-Chantelot C. (2013). Epidemiology of congenital neutropenia. Hematol./Oncol. Clin. N. Am..

[25] Carlsson G., Fasth A., Berglof E., Lagerstedt-Robinson K., Nordenskjold M., Palmblad J., Henter J.I., Fadeel B. (2012). Incidence of severe congenital neutropenia in Sweden and risk of evolution to myelodysplastic syndrome/leukaemia. Br. J. Haematol..

[26] Papadaki H.A., Xylouri I., Coulocheri S., Kalmanti M., Kafatos A., Eliopoulos G.D. (1999). Prevalence of chronic idiopathic neutropenia of adults among an apparently healthy population living on the island of Crete. Ann. Hematol..

[27] Andersen C.L., Tesfa D., Siersma V.D., Sandholdt H., Hasselbalch H., Bjerrum O.W., Felding P., Lind B., Olivarius Nde F., Palmblad J. (2016). Prevalence and clinical significance of neutropenia discovered in routine complete blood cell counts: A longitudinal study. J. Int. Med..

[28] Grann V.R., Bowman N., Joseph C., Wei Y., Horwitz M.S., Jacobson J.S., Santella R.P., Hershman D.L. (2008). Neutropenia in 6 ethnic groups from the Caribbean and the U.S. Cancer.

[29] Bernini J.C. (1996). Diagnosis and management of chronic neutropenia during childhood. Pediatr. Clin. N. Am..

[30] Yang S., Wu J., Ding C., Cui Y., Zhou Y., Li Y., Deng M., Wang C., Xu K., Ren J. (2017). Epidemiological features of and changes in incidence of infectious diseases in China in the first decade after the SARS outbreak: An observational trend study. Lancet Infect. Dis..

[31] Davgasuren B., Nyam S., Altangerel T., Ishdorj O., Amarjargal A., Choi J.Y. (2019). Evaluation of the trends in the incidence of infectious diseases using the syndromic surveillance system, early warning and response unit, Mongolia, from 2009 to 2017: A retrospective descriptive multi-year analytical study. BMC Infect. Dis..

[32] Palmblad J., Papadaki H.A. (2008). Chronic idiopathic neutropenias and severe congenital neutropenia. Curr. Opin. Hematol..

[33] Auner H.W., Klintschar M., Crevenna R., Beham-Schmid C., Hoefler G., Mitterbauer G., Linkesch W., Sill H. (1999). Two case studies of chronic idiopathic neutropenia preceding acute myeloid leukaemia. Br. J. Haematol..

[34] Papadaki H.A., Kosteas T., Gemetzi C., Alexandrakis M., Psyllaki M., Eliopoulos G.D. (2002). Two patients with nonimmune chronic idiopathic neutropenia of adults developing acute myeloid leukemia with aberrant phenotype and complex karyotype but no mutations in granulocyte colony-stimulating factor receptor. Ann. Hematol..

[35] Bhat R.Y., Varma C.P., Bhatt S. (2014). An infant with chronic severe neutropenia. BMJ Case Rep..

[36] Ikewaki J., Kawano R., Sato T., Imamura T., Kohno K., Ogata M., Ohtsuka E., Kadota J. (2012). An acquired CSF3R mutation in an adult chronic idiopathic neutropenia patient who developed acute myeloid leukaemia. Br. J. Haematol..

